# Optimization of mesoporous titanosilicate catalysts for cyclohexene epoxidation via statistically guided synthesis

**DOI:** 10.1007/s10853-018-2057-2

**Published:** 2018-01-29

**Authors:** A. S. Perera, P. Trogadas, M. M. Nigra, H. Yu, M.-O. Coppens

**Affiliations:** 1 Centre for Nature Inspired Engineering, University College London, Torrington Place, London, WC1E 7JE UK; 2 Department of Chemical Engineering, University College London, Torrington Place, London, WC1E 7JE UK; 3 Department of Chemical Engineering, University of Utah, Salt Lake City, UT 84112 USA

## Abstract

An efficient approach to improve the catalytic activity of titanosilicates is introduced. The Doehlert matrix (DM) statistical model was utilized to probe the synthetic parameters of mesoporous titanosilicate microspheres (MTSM), in order to increase their catalytic activity with a minimal number of experiments. Synthesis optimization was carried out by varying two parameters simultaneously: homogenizing temperature and surfactant weight. Thirteen different MTSM samples were synthesized in two sequential ‘matrices’ according to Doehlert conditions and were used to catalyse the epoxidation of cyclohexene with *tert*-butyl hydroperoxide. The samples (and the corresponding synthesis conditions) with superior catalytic activity in terms of product yield and selectivity were identified. In addition, this approach revealed the limiting values of each synthesis parameter, beyond which the material becomes catalytically ineffective. This study demonstrates that the DM approach can be broadly used as a powerful and time-efficient tool for investigating the optimal synthesis conditions of heterogeneous catalysts.

**Electronic supplementary material:**

The online version of this article (10.1007/s10853-018-2057-2) contains supplementary material, which is available to authorized users.

## Introduction

Synthetic porous titanosilicates are widely applied as active and selective heterogeneous catalysts to produce industrially relevant organic precursors [1–5]. Since their introduction in 1983, various categories, such as microporous [6–8], mesoporous [9–13] and layered types [14–19], have been synthesized. These target different applications, such as ion exchange [20–24], adsorption [25–28], membrane separation [29–32], pillaring templating [33, 34] and oxidation catalysis [35–38].

Despite the diversity and versatility of titanosilicates, there remain challenges in synthesizing the preferred structures. It is desirable to optimize synthesis procedures to facilitate materials development in a faster, more reproducible way [39]. The requirement of specialized conditions [40, 41] and high sensitivity to changes in synthesis conditions often results in lower than expected activity of catalyst candidates [42–44]. Hence, the use of a statistically guided approach could greatly benefit the optimization of titanosilicate synthesis. However, conventional optimization techniques, such as factorial, Plackett–Burman, central composite, Box–Behnken and orthogonal designs, have many disadvantages [45–47].

Factorial designs or Plackett–Burman designs, for example, are first-order models or univariate systems, where only one parameter can be studied at the same time for optimization [46]. Full factorial models can be used to investigate every factor at two levels. For a large number of factors, however, a fractional factorial model becomes necessary. The limitation of allowing only two factors to be investigated at any one time is disadvantageous, as many processes are complex, requiring multivariate systems in order to simultaneously and properly study the effects of several parameters. In such instances, second-order models, which involve more than two factors, such as central composite design (CCD), Box–Behnken design (BBD), Doehlert matrix design or orthogonal design (OD) can be used.

CCD has some advantages, as it requires fewer experiments than the full factorial, three-level designs [48]. It combines full or fractional factorial designs with additional star points and a centre point, and can fit quadratic polynomials, with rotatable or orthogonal properties. BBD is another second-order, rotatable model, that allows the proportional increase in the number of design points, to the number of polynomial coefficients, and is based on an incomplete factorial design with three levels [49].

OD is another well-known type of factorial design [50]. By definition, factor analysis becomes ‘orthogonal’ if multiple factors in an experiment can be analysed independently of each other. OD is very useful to analyse a small but representative sample from a large set of data, when analysing all data sets is not practical [51]. However, this technique is not always useful, particularly in material synthesis, when multiple experimental parameters have a combined significant effect on the final product formed, because they depend on each other.

The Doehlert matrix (DM) is also a second-order model, but has distinct advantages over factorial, CCD, BBD and OD models [45, 47, 52]. Doehlert designs are simpler and allow optimization of a process or reaction with fewer experiments, thus saving time and cost. They can be moved through the experimental space effectively, and results from previous experiments can be used to guide subsequent, adjacent experimental points (ESI, Figure S1) [53]. Also, they allow the user to adjust the range of each parameter, at each level of the related factors, especially if it is difficult to obtain a definite optimum point.

The DM approach becomes particularly advantageous, when the simultaneous effects of multiple experimental parameters become significant for optimizing a process. Synthesis conditions are often affected by fractions of parameter values; thus, the latter may need to be probed based on a range that contains fractions of the minimum and maximum of the range. The DM gives the investigator this freedom. In addition, the DM approach allows sequential investigation of regions in matrix space, based on previously obtained results. Thus, if a researcher recognizes a region of favourable reaction parameters, but not necessarily one optimal point, another matrix can be built around or in the direction of interest. Moreover, the range of experimental parameters can be narrowed down, if necessary, to enhance the focus around a region of interest (ESI, Figure S1). Hence, the DM approach has been widely utilized in analytical sciences, as an optimization tool and as a simple alternative to orthogonal design [47]. The method has been utilized successfully in oil refining, homogeneous catalysis, heavy metal analysis and food science, for optimization of experimental and analytical parameters [54–59]. ESI, Sects. 1 and 2, contains a more detailed discussion of the Doehlert matrix approach.

Herein, we introduce the use of the DM model as an efficient, versatile statistical optimization method, to investigate optimal synthesis conditions of titanosilicates. We demonstrate that this model is particularly desirable for a complex synthesis problem, such as the development of titanosilicates for epoxidation catalysis. Traditionally, the synthesis of titanosilicates has been carried out *via* techniques such as direct hydrothermal synthesis [60–62], the dry gel conversion method [63] and the post-synthesis method [64]. Later on, amorphous varieties became prominent, targeting bulky organic substrates and oxidants [39, 65], as opposed to the conventional, ordered, crystalline [66–68] types, which were suitable for smaller reactants. Soft templating techniques have been utilized to generate such amorphous titanosilicates along with uniform particle shape and size, and increased surface area and robustness [44, 45]. However, statistically guided optimization of the synthesis of titanosilicates is not widely reported.

The goal of this study was to develop a systematic approach to improve the catalytic activity of titanosilicates, *via* optimization of the synthesis conditions, utilizing the Doehlert matrix model. A mesoporous titanosilicate (MTSM) was synthesized, *via* surfactant templating, as an effective heterogeneous catalyst for the epoxidation of cyclohexene with TBHP as oxidant. The effect of the pore structure and the amount of framework Ti^4+^ on catalytic performance was also investigated.

## Experimental

### Synthesis of MTSM material

Synthesis was based on procedures that were modified from those published by Li and Coppens [39]. All chemicals were purchased from Sigma-Aldrich and used without further purification.

### General procedure

1.0 ml of Ti(IV) *n*-butoxide (99%) was added dropwise to 30.0 ml of DI water (18.2 MΩ), at 4 °C, under magnetic stirring, in order to form Ti(OH)_4_ precipitate. The precipitate was filtered under vacuum and washed with DI water. The Ti(OH)_4_ was then dissolved in 4.0 ml of 4 N HNO_3_ to produce TiO(NO_3_)_2_ active species. The TiO(NO_3_)_2_ was then mixed with a solution of 6.6 ml tetraethyl orthosilicate (TEOS, 98%) and 2.0 ml ethanol and stirred vigorously for 30 min, to form isolated Ti^4+^ sites in the silica matrix. The physical structure of this liquid mixture was then transformed into mesoporous microspheres by surfactant templating. The titanosilicate mixture was added to a mixture of 26.1 g kerosene and 7.9 g Span 80, and homogenized with an Ultra-Turrax homogenizer, at 3000 rpm for 2 h, at 80 °C. The microspheres formed were then vacuum-filtered and washed with acetone and DI water, followed by drying at 80 °C for 2 h. Finally, materials were calcined at 750 °C for 6 h.

### Characterization

FTIR spectroscopy was carried out with a Bruker Vertex 70 instrument. Raman spectroscopy was conducted with a Renishaw InVia Raman microscope using a Kimmon He/Cd laser at 442 nm wavelength. SEM images were taken using JEOL JSM-6480LV and JEOL JSM-5410LV scanning electron microscopes. EDX was performed using the above instruments in low vacuum mode. HRTEM images were taken with a JEOL 2100 microscope operating at 200 keV. The samples were dispersed in methanol and then dried on a Holey carbon film Cu grid, for TEM observation. Thermogravimetric analysis (TGA) was performed on a PerkinElmer TGA7, at 303–1273 K using dry air flow with a heating rate of 10 K/min. Powder X-ray diffraction (PXRD) patterns were obtained with a Stoe STADI-P instrument, using Cu Kα1 radiation operated at 40 kV and 30 mA. Nitrogen adsorption/desorption isotherms were conducted on a Quantachrome Autosorb iQ_2_, using the NLDFT method to evaluate surface area, pore volume and pore size distributions, from the adsorption branch of the isotherms. Diffuse reflectance (DR) UV–Vis data were obtained with an Agilent Technologies Cary 4000 UV–Vis Spectrophotometer with a Harrick Praying Mantis diffuse reflectance accessory. XPS spectra of samples 1–13 were recorded on a Thermo Scientific spectrometer with Cu Kα radiation. The analyser was set at a pass energy of 20 eV for high-resolution spectra of all the individual elements in each sample tested. Approximately 2–5 mg of each powder sample was mounted on a stainless steel sample holder. The background was determined using the Shirley-type background correction, and the curves were fitted with Gaussian and Lorentzian product functions.

### Doehlert matrix approach to optimize synthesis

**Figure 1:**
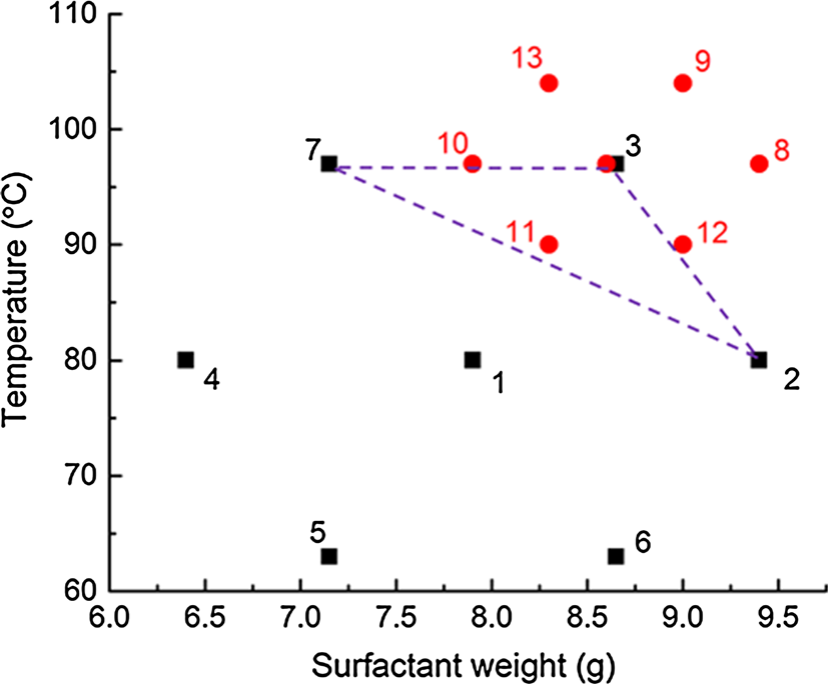
Samples 1–13, corresponding to Doehlert matrices 1 (DM1) and 2 (DM2). The triangle depicts the matrix region in DM1 that generated samples with high catalytic activity, which led to designing DM2

The synthesis conditions were optimized by changing relevant parameters in the synthesis procedure, according to conditions specified by the Doehlert matrix model. The first matrix (Table 1 and Fig. 1, samples 1–7) was designed by changing surfactant mass and temperature. Thus, a two-factor system was designed, with seven total data point sets, consisting of one centre point and six other points lying at corners of a regular hexagon (refer ESI Sects. 1 and 2). In this approach, the coordinates of each point are values for factor 1 and factor 2; hence, two parameters are changed simultaneously for each experiment. The centre point consists of reference experimental conditions. Next, the parameters that would be factor 1 and factor 2 have to be decided, since they contain five (i.e. 0, 1, − 0.5, − 1, 0.5) and three (i.e. 0, 0.866, − 0.866) values, respectively. In order to determine this, an understanding of which of these parameters would have a stronger impact on the final outcome of the study, in this case, catalytic performance is necessary. The experience and prior knowledge of the researcher play a critical role here. Based on our understanding of factors that affect chemical and physical properties of the catalyst, we chose the surfactant mass as factor 1, with five values. The parameter with less expected impact, i.e. homogenizing temperature, was chosen as factor 2, with three values. The centre point corresponded to 7.9 g of surfactant and 80 °C for the homogenizing temperature [39]. Exact values of factors 1 and 2 were calculated based on Eq. 1, given by the Doehlert model:

1 $$ P_{i} = x + a*F_{i} $$

where *P*_*i*_ = calculated value of parameter, *x* = starting value of parameter, *a* = probe limit of parameter, *F*_*i*_ = value of factor (coded) for experiment *i, i* = experiment number.

**Table 1:** Doehlert matrices 1 (DM1) and 2 (DM2)

Sample	Factor 1	Surfactant mass/g	Factor 2	Temperature/°C
1	0	7.9	0	80
2	1	9.4	0	80
3	0.5	8.6	0.866	97
4	− 1	6.4	0	80
5	− 0.5	7.2	− 0.866	63
6	0.5	8.6	− 0.866	63
7	− 0.5	7.2	0.866	97
8	1	9.4	0	97
9	0.5	9.0	0.866	104
10	− 1	7.9	0	97
11	− 0.5	8.3	− 0.866	90
12	0.5	9.0	− 0.866	90
13	− 0.5	8.3	0.866	104

Change in surfactant mass and temperature

The experiments were later extended to design a second matrix (samples 8–13) with a narrower range of conditions. Limits for the first matrix were ±1.5 g for surfactant mass and ±17 °C for temperature. For the second matrix, limits were reduced to ±0.8 g and ±7 °C, respectively. These parameters, their starting values and limits were chosen based on preliminary experiments, which indicated them to be the most influential features on the products formed [47]. A third matrix was designed by changing TEOS concentration and homogenizing temperature.

### Catalytic experiments

MTSM samples were catalytically characterized *via* epoxidation of cyclohexene with TBHP according to a procedure adapted from the literature [65]. 25 mmol of cyclohexene was mixed with 20 mL of decane as solvent in a batch reactor, followed by 100 mg of MTSM catalyst. Next, approximately 7 g of 4 Å molecular sieves were added to the reactor in order to remove moisture from the mixture. The mixture was kept under argon for 30 min to create an inert atmosphere and stirred at 60 °C for another 30 min. 5.5 mmol of 5.5 M TBHP solution in decane was then added to initiate the reaction (ESI Scheme 1). Samples from the reaction mixture were withdrawn at relevant time periods and analysed by gas chromatograph using a GC-2014 Shimadzu Gas Chromatograph, employing a ZB-WAXplus Zebron capillary GC column.

Calibration curves for TBHP, cyclohexene and cyclohexene oxide were obtained using standard solutions. These were used for the determination of the relevant concentrations of the reaction mixtures, *via* GC analysis (ESI Figures S4, S5, S6 and S7).

### Kinetic studies

Kinetic experiments were conducted on the most promising sample from the study, i.e. sample 7. This sample displayed the highest product yield and selectivity with excellent reagent conversion, during a 24-h reaction. Further epoxidation experiments were performed on this catalyst, and samples were taken out at time intervals 0, 2, 4, 6, 8 and 24 h, and immediately subjected to GC analysis.

## Results and discussion

### Criteria behind synthesis optimization experiments

The parameters of the MTSM microspheres were probed according to two routes: (1) change in morphology, i.e. particle size, shape and pore network properties, and (2) change in chemical structure, i.e. ratio of Ti to Si. The former was achieved by changing the amount of surfactant added during templating, as well as the reaction temperature, and the latter by changing the amount of TEOS added during synthesis and the reaction temperature. Note that, in each case, two synthesis parameters were changed simultaneously. Two matrices (DM1 and DM2) were designed for route 1.

The first Doehlert matrix (DM1) was designed as a two-factor system, around surfactant mass and homogenizing temperature values known from the literature [39], as primary and secondary variables, respectively (Table 1, samples 1–7 and Fig. 1). Using the relevant multiplication factors given by the Doehlert model, six other synthetic conditions were calculated, and seven samples in total were synthesized and used as catalysts for the epoxidation of cyclohexene using TBHP as oxidant (Table 2, samples 1–7). Cyclohexene was in excess and TBHP was the limiting reagent with a molar ratio of 5:1.

**Table 2:** Results of cyclohexene epoxidation with TBHP using MTSM as catalyst after 24-h reaction in batch

Doehlert matrix number	Sample	Cyclohexene conversion %^a^ (±4)	TBHP conversion %^b^ (±8)	Epoxide yield w.r.t TBHP %^c^	Selectivity %^d^
DM1	1	7	83	56	65
	2	12	94	53	55
	3	17	97	66	68
	4	5	69	26	35
	5	4	65	10	15
	6	8	79	30	38
	7	12	95	77	81
DM2	8	8	96	47	49
	9	17	96	49	51
	10	15	95	54	57
	11	10	97	49	50
	12	13	93	48	52
	13	11	97	54	56

Refer to ESI, Sect. 6 for details on calculations for a, b, c and d

Based on the results obtained in terms of the yield and selectivity of the desired product formed with respect to the limiting reagent (i.e. amount of epoxide formed with respect to TBHP), a region of high catalytic activity was identified. This included samples 2, 3 and 7, where sample 7 was the clearly superior catalyst (Fig. 1). Based on these, a second matrix (DM2) was designed to further investigate the region of high activity (samples 8–13, Fig. 1 and Table 2). The range of synthesis conditions for DM2 was narrowed down for optimal investigation of the relevant parameter region (Fig. 1). Although DM2 had reasonably high catalytic activity, the results of product yields and selectivity did not surpass those of DM1 sample 7; hence, sample 7 was considered as the optimal point and no further matrices were designed for route 1.

Overall, sample 7 has much improved catalytic activity in terms of reagent conversion, product yield and selectivity compared to the original starting material, sample 1. Sample 3 is the second-best catalyst, followed by sample 2, which gives similar yield and selectivity to sample 1. Samples 4, 5 and 6 can be identified as poor catalysts. This shows that utilization of the DM approach has been successful in significantly enhancing the catalytic activity of the MTSM material, using a relatively small number of experiments. Additionally, samples 8–13 exhibited similar catalytic activity with respect to each other. This means that the synthesis conditions could be altered within a certain range without compromising the product yields and selectivity of titanosilicates. Such findings are significant, especially in designing large-scale synthesis procedures, where high product formation is expected with minimum raw material and energy costs. Doehlert matrix studies could similarly guide the industrial-scale design of synthesis processes.

A second route to optimize the synthesis *via* changing the chemical structure was accomplished by means of a third Doehlert matrix, DM3 (ESI Table S4 and Figure S8). Here, TEOS concentration and homogenizing temperature were probed as primary and secondary changing parameters, respectively. The results of DM3 did not surpass those of DM1 in a significant manner. Therefore, no further experiments were conducted along this route.

### Characterization of MTSM

**Figure 2:**
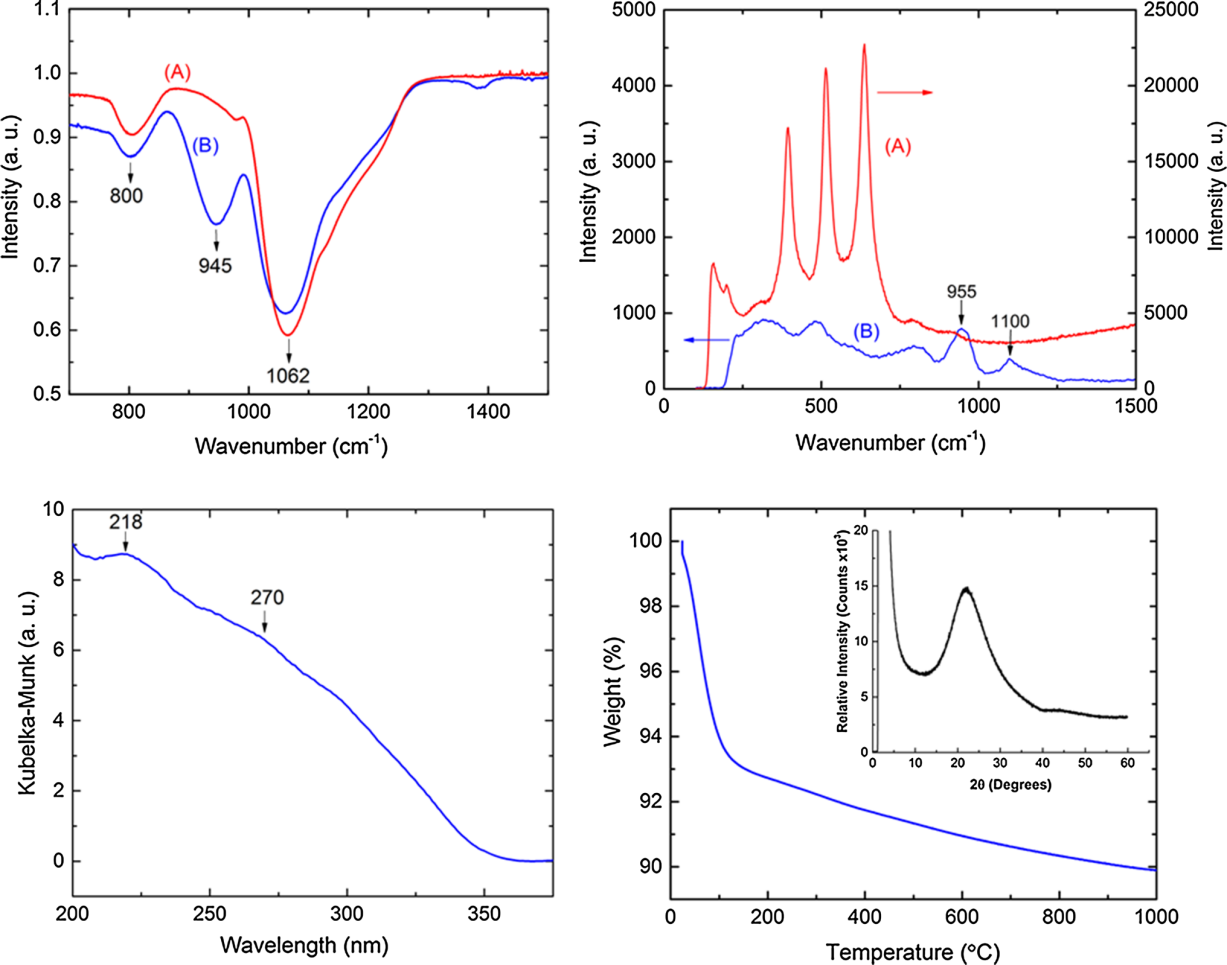
Characterization of MTSM sample 7. Top left—FTIR spectra of **a** SiO_2_ and **b** MTSM showing characteristic Ti-O-Si peak at 945 cm^−1^, top right—Raman spectra of **a** Commercial anatase TiO_2_ and **b** MTSM, showing characteristic peaks at 955 and 1100 cm^−1^, bottom left—diffuse reflectance UV–Vis, indicating the presence of isolated tetrahedral Ti at 218 nm and Ti–O–Ti oligomers at 270 nm. Bottom right—TGA shows a mass loss at 30–140 °C, indicating loss of water, inset—PXRD data showing one broad peak, indicating that the material is amorphous

Initial characterization of MTSM was performed with FTIR, Raman and DR–UV in order to measure the presence of the isolated Ti–O–Si species that are important in catalysis. In the FTIR spectra, the characteristic peak at 945 cm^−1^, corresponding to the Ti–O–Si asymmetric stretching mode, was observed [69, 70] (Fig. 2—top left). The large peak at 1062 cm^−1^ corresponds to the antisymmetric Si–O–Si stretching mode and the peak at 800 cm^−1^ corresponds to the O–Si–OH bending mode, as compared with SiO_2_ [69]. The Raman spectrum also shows the characteristic Ti–O–Ti bands at 955 and 1100 cm^−1^ for the symmetric and the antisymmetric stretch, respectively (Fig. 2—top right) [71, 72].

Characteristic TiO_2_ bands were observed for commercial anatase at 150, 395, 515 and 640 cm^−1^, whereas these were absent in the MTSM sample. DR–UV clearly indicated the presence of a band at 218 nm, confirming the presence of isolated Ti^4+^ in tetrahedral geometry [72] (Fig. 2—bottom left). However, a broad shoulder at 270 nm was also observed, indicating that there is considerable amount of bulk TiO_2_ in the material [73]. TGA was conducted to examine the stability of the synthesized MTSM and shows only one mass loss event between 30 to 140 °C, resulting in a weight loss of approximately 6% (Fig. 2—bottom right). This could be attributed mainly to the loss of adsorbed water. A further gradual loss of mass was observed that continued until 1000 °C, and could be due to loss of residual kerosene. The MTSM material appeared to be thermally stable and retained about 90% of its total weight, even at 1000 °C.

**Figure 3:**
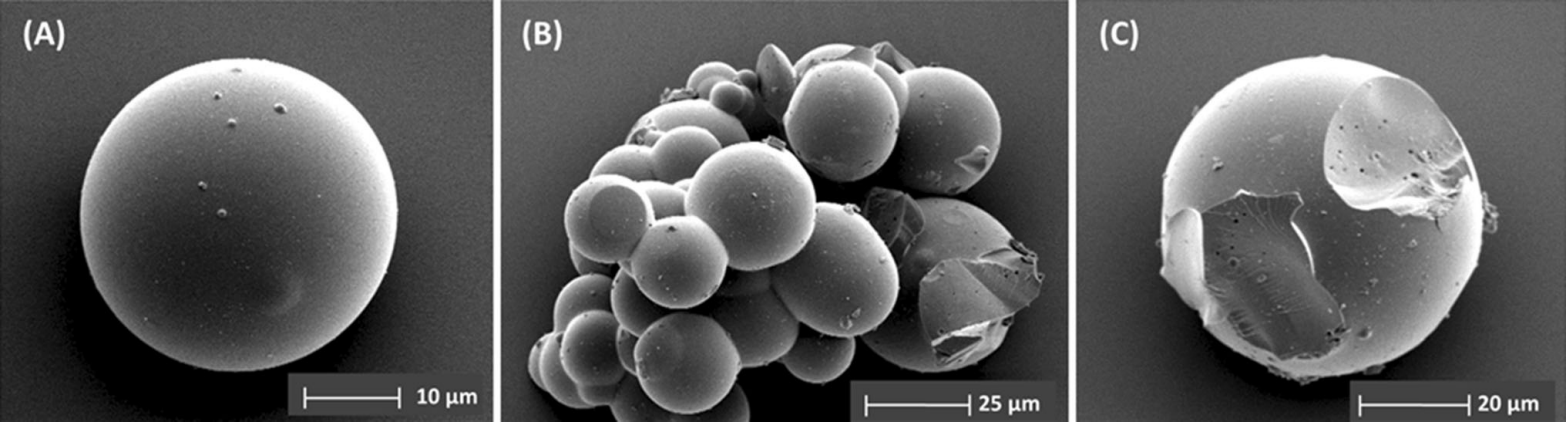
SEM images of MTSM microspheres, after calcination at 750 °C for 6 h: **a** an individual sphere; **b** cluster of spheres fused together, indicating average sizes of 20–30 µm; **c** a crushed microsphere, revealing that the material is mostly solid inside, with a few macropores from the surfactant oil emulsion, which was calcined

Imaging techniques were used to investigate the morphology of the synthesized MTSM samples. SEM images revealed that the material was of microspherical morphology (Fig. 3). These were predominantly 20–30 µm in diameter and were mostly fused together. The microspheres appeared to be solid; however, some of them contained visible pores of 1–3 µm, possibly from trapped surfactant and oil, which was removed during calcination. Note that these spheres are not hollow as in the method described by Li and Coppens [39], due to minor changes in synthesis procedure; as the reactions are not diffusion limited in microspheres, a hollow interior serves no particular purpose for the epoxidation reaction. EDX elemental analysis revealed the particular purpose for the epoxidation reaction. EDX elemental analysis revealed that the material was only composed of Si, O, Ti and residual C (ESI Table S5, Figure S9). No other elements were present in any significant amount. Furthermore, HRTEM images of the crushed material indicated a predominantly amorphous morphology with small crystalline regions (ESI Figure S10). The amorphous nature of the material was confirmed by PXRD experiments, where a single broad peak was observed with no indication of any sharp peaks (Fig. 2—bottom right—inset).

### Determining the factors that affect catalytic activity

We found that changing the synthetic conditions systematically and simultaneously was an effective way to find conditions that generate significantly enhanced catalytic activity and selectivity of the MTSM material (Tables 1 and 2). A combination of higher temperatures and moderate surfactant amounts led to the synthesis of more effective catalysts. Sample 7, for example, gave 38 and 25% higher product yield and selectivity, respectively, compared to the starting point (Table 2, sample 1 versus 7). However, high surfactant concentrations and temperatures became ineffective beyond certain limits. This was evidenced by the catalytic activity results of the matrix DM2 (Table 2, samples 8–13).

**Figure 4:**
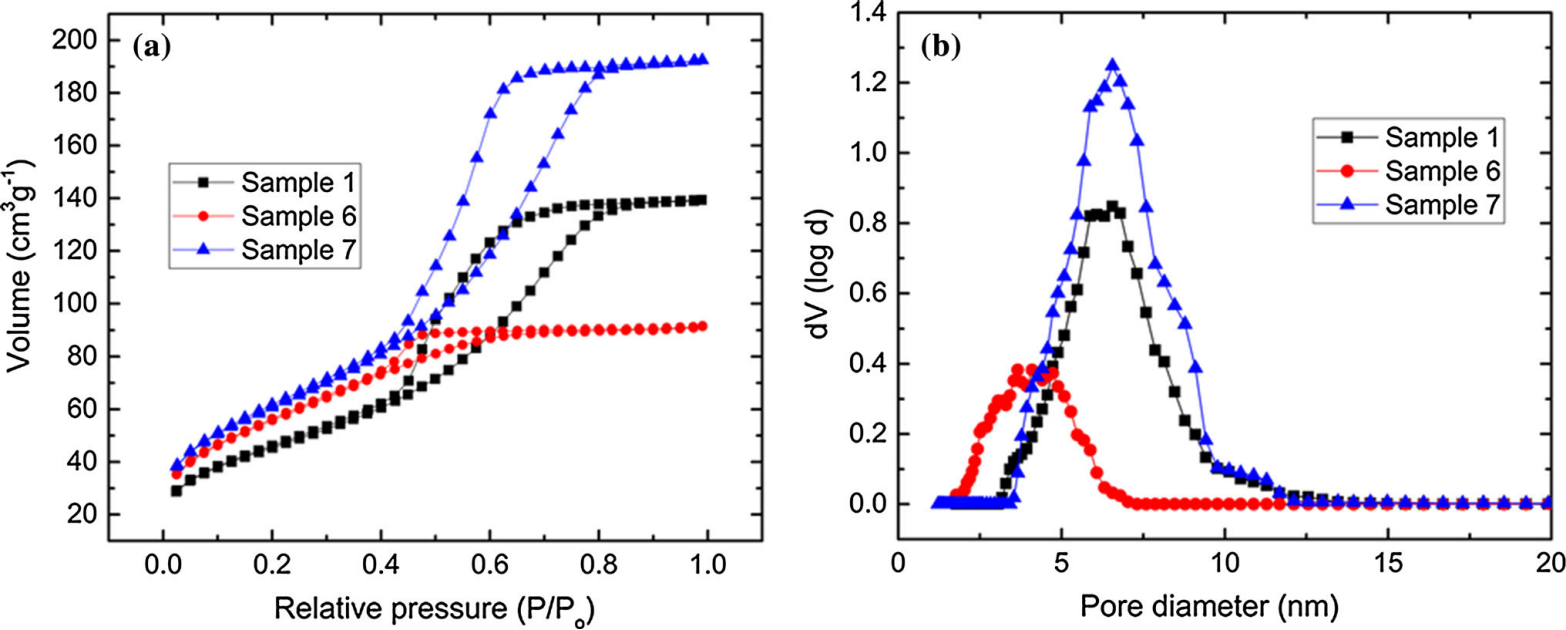
**a** Adsorption–desorption isotherms for samples 2, 3 and 7, using N_2_ at 77 K. **b** Pore size distributions for samples 2, 3 and 7, calculated using the NLDFT method

**Figure 5:**
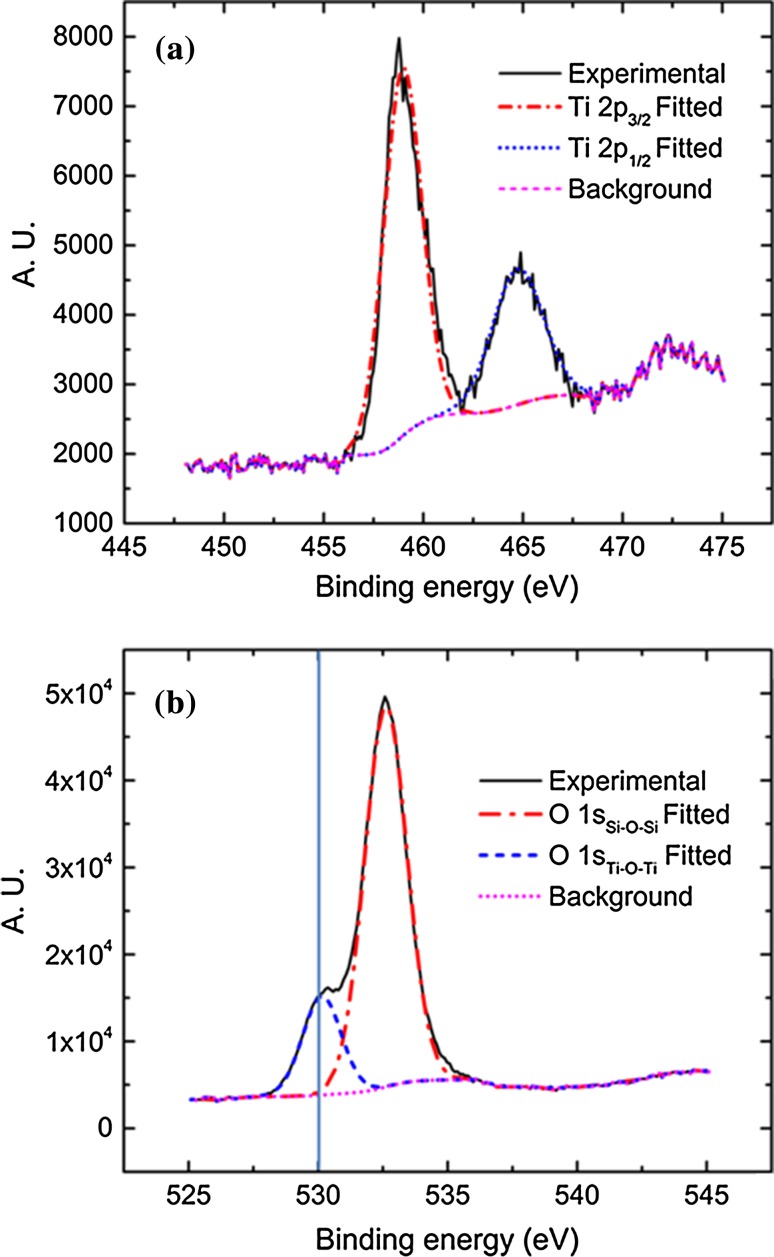
**a** XPS O 1 s spectra of sample 7, before and after reaction. **b** XPS O 1 s spectra for sample 7. Vertical line indicates position of pure TiO_2_ (530 eV)

The two predominant factors that affected the catalytic activity of porous titanosilicates were the number of isolated Ti^4+^ sites per unit weight and the nature of the pore network [74]. Nitrogen physisorption (Fig. 4) and XPS measurements (Table 3 and Fig. 5) were conducted on samples 1–7, in order to investigate these factors thoroughly. BET analysis revealed a direct relationship between MTSM properties (i.e. surface area, pore size distribution, pore volume) and catalytic activity. The highest surface area, pore volume and mesopore size were associated with the best catalyst, sample 7. The samples with high to moderate surface areas and pore volumes and pore sizes, such as 3, 2 and 1, had good to moderate catalytic activity, accordingly. Samples 5 and 6, which were clearly poor catalysts, had much smaller pore diameters, limiting access to the catalytic active sites by the bulky cyclohexene and TBHP reagents.

**Table 3:** Nitrogen physisorption and XPS data for samples 1–7

Sample	BET surface area /m^2^ g^−1^	Total pore volume/cm^3^ g^−1^	Micropore volume/%	Average pore diameter/nm	Tetrahedral Ti 2*p*_1/2_/%
1	166	0.208	6	6	0.74
2	168	0.183	12	5	1.15
3	142	0.165	6	6	1.7
4	177	0.186	16	5.5	0.83
5	–	–	–	2.5	0.46
6	180	0.126	60	4	1.43
7	231	0.292	4	9	1.09

Furthermore, sample 6 had the smallest pore volume out of which 60% were micropores. Surface area and pore volume for sample 5 (which was the worst catalyst) could not be calculated as the isotherms did not close (ESI Figure S11). Sample 4, despite having a high surface area and pore volume, was a rather poor catalyst, possibly due to having a large micropore fraction (16%), which hindered accessibility of reagents to the active sites.

XPS measurements were conducted on all samples of DM1 (samples 1–7), in order to investigate the effect of the amount of active tetrahedral Ti^4+^ per unit volume on their catalytic activity (Table 3 and ESI Table S2). The XPS Ti 2p spectra were deconvoluted into Ti 2p_3/2_ and Ti 2p_1/2_ peaks at 459 and 465 eV, respectively, with constrained 2:1 intensity ratio [75, 76]. The two peaks occur due to multiplet splitting from different spin distributions of the electrons of the Ti band structure [77, 78]. The two Ti 2p peaks indicated that Ti^4+^ was the only oxidation state of the MTSM material (Fig. 5a) and represent Ti^4+^ in octahedral (lower binding energy) and tetrahedral (higher binding energy) coordination [79–81]. The catalytically active, isolated Ti–O–Si sites were represented by the tetrahedral coordination (Ti 2p_1/2_), whereas the octahedral coordination accounts for the inactive Ti–O–Ti oligomers and aggregated TiO_2_. Evidence of such Ti–O–Ti oligomers was found during diffuse reflectance UV–Vis experiments, with the presence of a weak shoulder at 270 nm [82, 83] (Fig. 2—bottom left).

The O 1*s* spectra provided information about the different chemical environments of oxygen in the MTSM. However, the framework Ti species in the tetrahedral Ti–O–Si environment were impossible to identify versus the Ti–O–Ti, as the former were masked by the Si–O–Si peak (Fig. 5b). The shoulder at ~531 eV generally corresponds to non-framework TiO_2_ species in octahedral environment [80, 81, 84]. The characteristic peak for TiO_2_, however, appeared at 530.1 eV, but the analogous peak of the synthesized MTSM was shifted by ~1 eV and appeared at ~531 eV. Such deviation towards higher binding energies suggested the presence of tetrahedral Ti–O–Si species [79, 80].

This was confirmed by the shift in binding energy for the Ti 2p spectra [80, 84]. The Si–O–Si peak of the silica framework appeared at 533 eV and was analogous to that of SiO_2_ (α-quartz) [84].

For samples 1–7, it was evident that the samples with high catalytic activity such as 2, 3 and 7, contain higher amounts of tetrahedral Ti^4+^ (Table 3). However, the effect is not linear, as poor catalysts, such as sample 6, contain high amounts of tetrahedral Ti^4+^ active sites, but these are not accessible to the reagents, due to the presence of large amounts of micropores. Clearly, a combination of high surface area, pore volume, pore size and isolated Ti^4+^ amount is necessary to bring about increased catalytic activity. The Doehlert matrix approach allowed exploration of synthetic conditions that lead to such desirable properties, in a relatively short time frame.

**Fig. 6:**
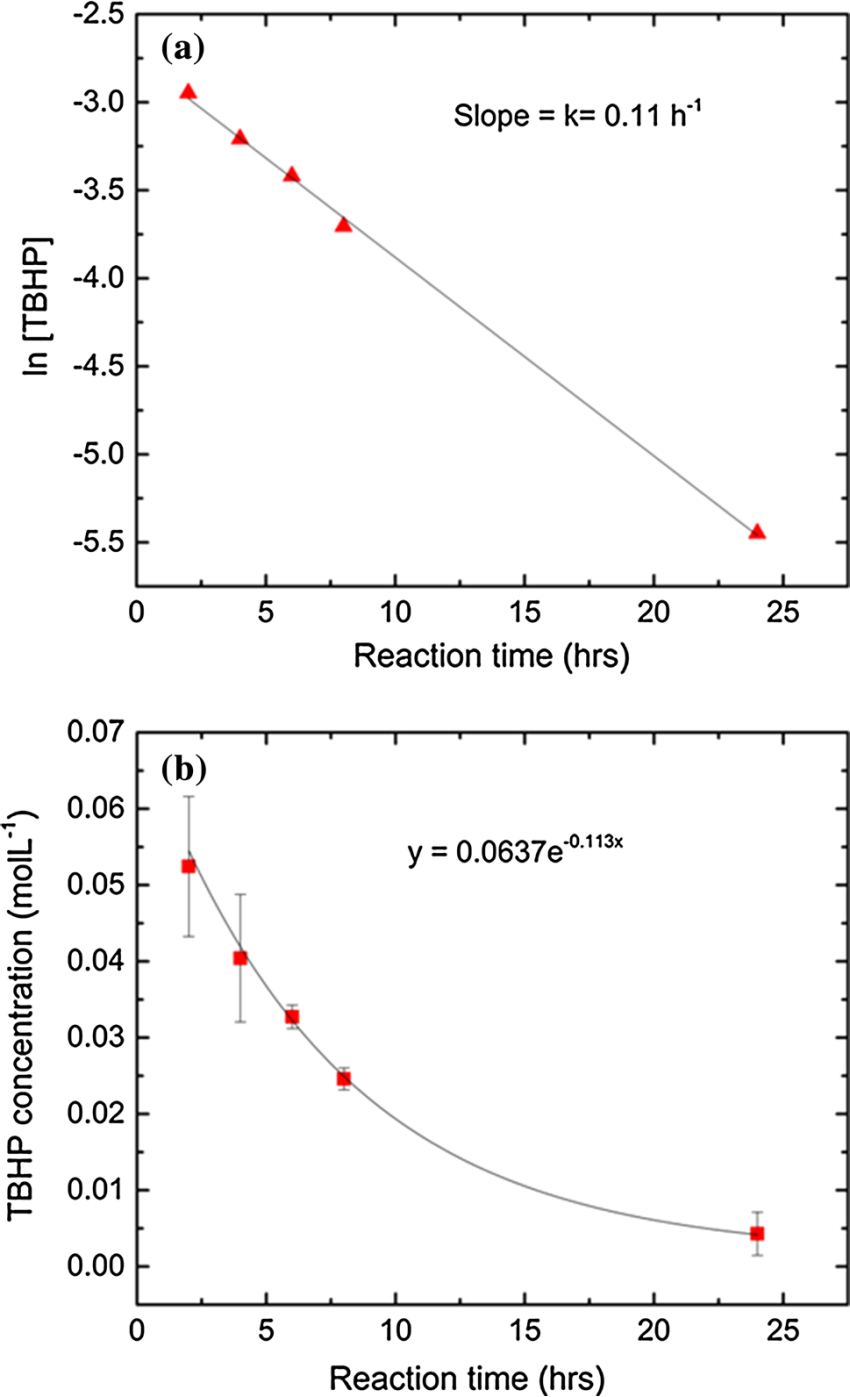
Kinetic experiments for sample 7. **a** ln[TBHP] vs reaction time. **b** TBHP concentration vs reaction time. Kinetics for, at least, 2–24 h are effectively first order

Kinetic experiments were conducted for the best catalyst, sample 7, in order to determine reaction order and rate coefficient. Samples were analysed at 0, 2, 4, 6, 8 and 24 h. A plot of ln[TBHP] *vs.* time for the 2–24 h period appeared linear (Fig. 6a), indicating that the reaction follows, effectively, first-order kinetics after a short initiation time [85]. The conversion of the limiting reagent TBHP with time decayed exponentially during this period (Fig. 6b), also in accordance with first-order kinetics. However, for the 0–2 h time period, a more rapid TBHP conversion was observed. The effective rate constant, *k*, for 2–24 h can be determined from the slope of the linear drop of ln[TBHP] *vs.* time and was found to be 0.11 h^−1^. Turnover frequency (TOF) values with respect to TBHP conversion were calculated for the reaction times of 2–24 h, based on the instantaneous reaction rates of TBHP and the concentration of Ti active sites (Eq. 2): [86, 87]

2 $$ {\text{TOF}} \,\left( {h^{ - 1} } \right) = \frac{{{\text{Instantaneous}}\,{\text{reaction }}\,{\text{rate}}}}{{\left[ {{\text{Ti}}\,\,{\text{active sites}}} \right]}} $$

The relevant weight % of Ti was calculated from the atomic % obtained from XPS, while the slope of the tangent line at each reaction time from Fig. 6b was used to find the instantaneous reaction rate. TOF is represented according to two ways: using the amount of tetrahedral Ti active sites and the total amount of Ti (Table 4). The reason for calculating two types of TOF values is to distinguish the results between catalytically active Ti 2*p*_1/2_ sites and the total amount of Ti, which also includes TiO_2_, i.e. the inactive Ti 2*p*_2/3_. The use of tetrahedral Ti is a better representation of the catalytic activity of the MTSM material, since DR–UV revealed the presence of significant amounts of TiO_2_ (Fig. 2—bottom left). The TOF values indicate that the reaction is reasonably fast, in comparison with other non-functionalized titanosilicates, used under similar conditions, for epoxidation of cyclohexene with TBHP [88–91]. ICP-AES experiments were conducted on samples 1–7 to evaluate the bulk Ti content in the materials (ESI, Table S2). Unlike XPS, ICP reveals the weight % of Ti. However, it does not reveal the oxidation or coordination states of different Ti species and therefore cannot distinguish between the amounts of active and non-active species. Therefore, tetrahedral Ti atomic % given by XPS was chosen to normalize the data, as it best represents the active species of MTSM.

**Table 4:** TOF values calculated using the tetrahedral Ti content of sample 7, with respect to TBHP conversion for different reaction times

Reaction time/h	TOF based on tetrahedral Ti/h^−1^	TOF based on total Ti/h^−1^
2	156	52
4	125	42
6	99	33
8	79	26
24	13	4

It is evident from the above discussion that using a statistically based modelling approach to guide the optimization of the synthesis conditions of titanosilicates brings about materials with superior catalytic activity, in a relatively easy way. It also reveals important structure–function relationships. Surfactant templating is shown to be an effective technique for generating mesoporous catalyst structures. The catalytic activity of the MTSM material is shown to be dependent upon this unique structure, along with its chemical composition. More detailed insights into the active site could be obtained in the future by *operando* studies, EXAFS and XANES measurements [92].

## Conclusions

We have successfully demonstrated the implementation of the Doehlert matrix approach for optimizing the synthesis of titanosilicates that leads to improved catalytic activity, achieved *via* simultaneously changing two synthesis parameters, which are homogenizing temperature and surfactant weight. Extensive characterization of the MTSM materials revealed that a complex combination of factors affects the catalytic activity. These include physical parameters, such as pore size distribution and micro-mesoporosity, together with chemical parameters, such as the amount of framework Ti^4+^. Kinetic experiments revealed that the catalyst has first-order kinetics between 2–24 h. The sample synthesized by homogenizing at 97 °C, with 7.2 g of surfactant, generated the best catalyst, with a high surface area and high accessible mesoporosity, and resulted in 95% conversion of TBHP and 81% selectivity towards epoxide. Understanding such intricacies that relate chemical structure and morphology to catalytic activity provides critical information leading to further progress in the field of titanosilicate catalysis. The Doehlert matrix optimization method thus enables the discovery of new and effective catalysts by efficiently and simultaneously probing synthesis parameters.

## Supplementary information

Electronic supplementary information is available free of charge at http://link.springer.com/. Advantages of using the Doehlert matrix model compared to selected other statistical models; Doehlert matrix experimental design; reaction scheme of cyclohexene with TBHP under experimental conditions; calibration curves for TBHP, cyclohexene and cyclohexene oxide; gas chromatogram of reaction mixture; details on calculations for catalytic experiments in Table 2 (calculation of reagent conversions, product yield, selectivity); XPS and ICP data; details of calculation of turnover frequencies; Doehlert matrix experiments for chemical structure modification: change in TEOS concentration and temperature; EDX experimental details, elemental analysis, image and spectrum; HRTEM imaging of catalyst; BET surface area of samples 2, 3, 4 and 5.

## Electronic supplementary material

Below is the link to the electronic supplementary material.

Supplementary material 1 (DOCX 2057 kb)

## Abbreviation

TBHP: Tert-butyl hydroperoxide
TEOS: Tetraethyl orthosilicate
DM: Doehlert matrix
FTIR: Fourier transform infrared spectroscopy
DR–UV: Diffuse reflectance ultraviolet–visible spectroscopy
SEM: Scanning electron microscopy
TGA: Thermogravimetric analysis
PXRD: Powder X-ray diffraction
HRTEM: High-resolution transmission electron microscopy
NLDFT: Non-local density functional theory
BET: Brunauer–Emmett–Teller theory
BJH: Barrett–Joyner–Halenda theory
EDX: Energy-dispersive X-ray spectroscopy
XPS: X-ray photoelectron spectroscopy
H_2_O_2_: Hydrogen peroxide
ICP-AES: Inductively coupled plasma–atomic emission spectroscopy
