# Supplementary material for: Optimization of mesoporous titanosilicate catalysts for cyclohexene epoxidation via statistically guided synthesis
Source: J Mater Sci. 2018 Jan 29;53(10):7279–93. doi: 10.1007/s10853-018-2057-2 (PMC6566288; doi:10.1007/s10853-018-2057-2)
Supplement: Supplementary file 1 — Supplementary material 1 (DOCX 2057 kb) [file 10853_2018_2057_MOESM1_ESM.docx]

**Supporting Information**

**Journal of Materials Science**

Optimization of Mesoporous Titanosilicate Catalysts for Cyclohexene Epoxidation via Statistically Guided Synthesis

A. S. Perera,^*^ ^a, b^ P. Trogadas, ^a, b^ M. M. Nigra,^b,c^ H. Yu,^b^ and M.-O. Coppens^* a, b^

^a^ Centre for Nature Inspired Chemical Engineering, University College London, Torrington Place, London, WC1E 7JE, UK

^b^ Department of Chemical Engineering, University College London, Torrington Place, London, WC1E 7JE, UK

^c^ Department of Chemical Engineering, University of Utah, Salt Lake City, Utah 84112, USA

1. **Advantageous features of the Doehlert matrix model**


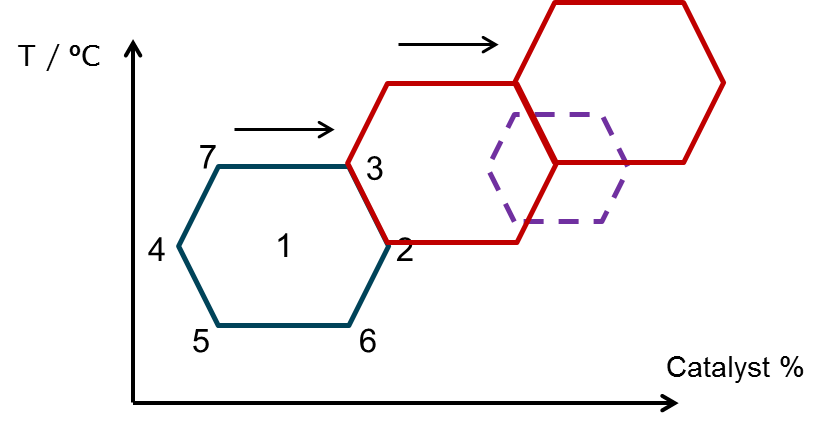


**Figure S1.** Doehlert matrix designs can "move in space" or be narrowed down, to focus on experimental regions of interest. The blue hexagon represents the original matrix experiments. Red hexagons represent experiments that are based on results of the original matrix. The purple matrix (dashed line) has narrower parameter limits.

**Note:** Orthogonal design is more suitable when a large number of parameters needs to be screened. It also allows the most significant factors to be identified, along with their relationship to each other. The Doehlert matrix approach is more suitable when significant factors have already been identified by preliminary research. The Doehlert approach can also be used in combination with other statistical models, such as factorial design, when features of different techniques become advantageous in experimental analysis.[^1^](#_ENREF_1)

1. **Doehlert matrix experimental design**

The Doehlert matrix model was introduced by D. H. Doehlert, as a facile method to generate uniformly spaced points over an experimental domain of interest.[^2^](#_ENREF_2) Herein, the method is demonstrated using two factors: If *x_j_* is the level of the *j*^th^ factor, an experiment with two factors can be represented as a two coordinate point (*x_1_*, *x_2_*). We can consider three such two-space points, and represent them as the three points of an equilateral triangle (Figure S2 (A)). Then, if we set the length of each side as 1, the value of h can be calculated using Pythagoras’ theorem: 1^2^ = 0.5^2^ + h^2^, and h is solved to be 0.5√3, which is, approximately, 0.866. Now, the three sets of coordinates of the triangle can be written as:

(0.000, 0.000),

(1.000, 0.000),

(0.500, 0.866), thus forming a two-space, regular simplex (Figure S2 (B)).

| 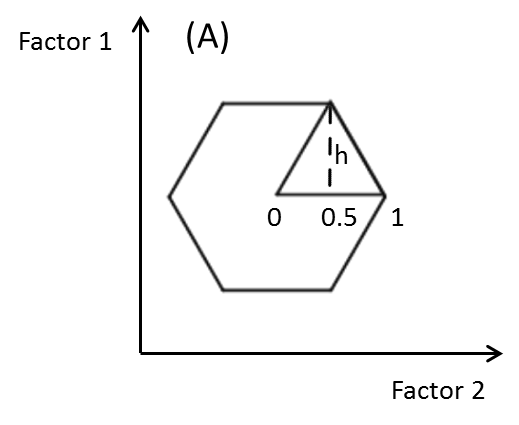 | 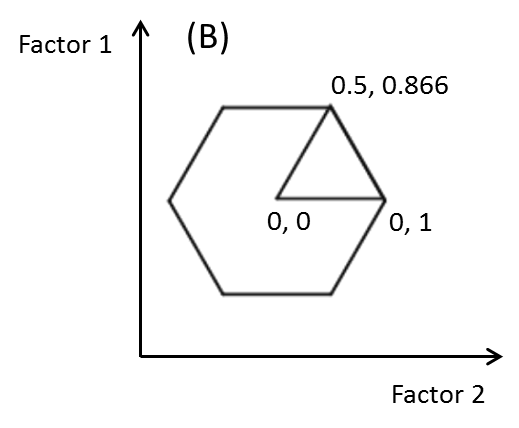 |
| --- | --- |

**Figure S2.** (A) - The two-spaced points of an equilateral triangle, represented inside a hexagon, (B) Coordinates calculated for an equilateral triangle, where the length of each side is 1.

The other points of the hexagon can be calculated by classical geometry to be:

(-1.000, 0.000),

(-0.500, -0 .866),

(-0 500, 0.866),

(0.500, -0.866).

Thus, we obtain seven data point sets in total, the first one for the centre and the six others for the corners of a regular hexagon, all distributed uniformly in space (Figure S3).

This method can be extended to three, four, five or more factors. If *d* is the number of factors, any regular simplex would form (*d*+1) points, one of which is the point of origin or centre point. The other points can be thought of as lying around the centre point in a sphere which has a radius of 1.0. If each of these points on the sphere is subtracted from the other *d* points in the simplex, another new *d* number of points will be generated. Thus, the total number of points would be *d*^2^+*d*+1, one being the centre point and the other *d*(*d*+1) points lying on a sphere of radius 1.0. For example, for a two-factor system, the total number of experiments will be 2^2^+2+1 = 7, and for a three-factor system, 3^2^+3+1 = 13 etc. Since this design generates uniformly spherical shells, Doehlert called these “uniform shell designs".

For our study, we chose to change two experimental parameters, *i.e.*, surfactant mass and homogenizing temperature (Table 1 in paper). Thus, it is a two-factor system, with seven total data point sets, consisting of one centre point and six other points lying at corners of a regular hexagon, as described earlier. Each point has two values for factor 1 and factor 2, hence, we can change two experimental parameters simultaneously for each experiment. The centre point consists of known experimental conditions, in this case 7.9 g of surfactant and 80 ˚C of homogenizing temperature. Now, we need to decide which parameters would be factor 1 and factor 2, since they have five (*i.e.*, 0, 1, -0.5, -1, 0.5) and three (*i.e.*, 0, 0.866, -0.866) values, respectively. In order to determine this, we need to understand which of these parameters would have a stronger impact on the final outcome of the study: in this case, catalytic performance. The experience and prior knowledge of the researcher plays a critical role here. Based on our understanding of factors that affect chemical and physical properties of the catalyst, we chose the surfactant mass as factor 1. This is because factor 1 has five values. The parameter with less expected impact, *i.e.*, homogenizing temperature, was chosen as factor 2, with three values.

The next step is to determine the probe limits for factors 1 and 2. These are, again, determined by the researcher, and are within a reasonable range of experimental conditions, for the relevant parameters. We chose our probe limits to be ±1.5 g for the surfactant mass and ±17 ˚C for the homogenizing temperature (Figure S3).


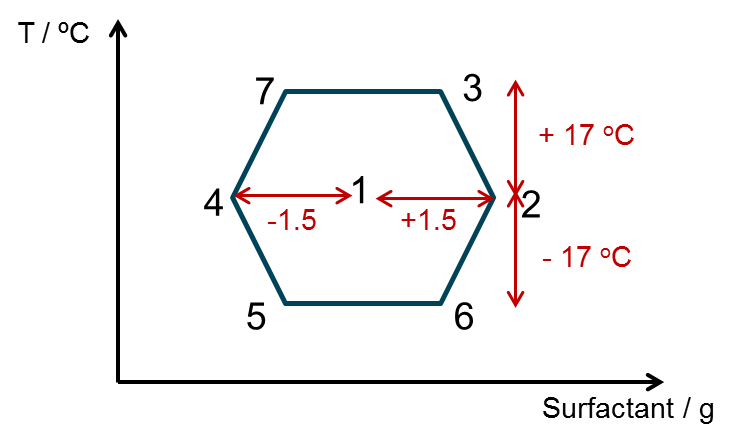


**Figure S3.** Probe limits for factors 1 and 2 for the seven experiments in a two-factor Doehlert matrix design.

The centre point is usually represented by number 1 and the other points as numbers 2-7, each being an arbitrary point of the hexagon. Using the following equation, individual values for the experimental parameters for factor 1 and 2 can be determined:

P*_e_* = x + a * F*_e_*

where:

P*_e_* = calculated value of parameter

x = starting value of parameter

a = probe limit of parameter

F*_e_* = value of factor (coded) for experiment *e*

*e* = experiment number

The values for the surfactant mass and homogenizing temperature, according to factors 1 and 2 of the Doehlert matrix, are calculated as shown in Table S1 below.

**Table S1.** Doehlert matrix 1 (DM1): Change of surfactant mass and temperature.

| **Sample** | **Factor 1** | **Surfactant mass / g** | **Factor 2** | **Temperature / ⁰C** |
| --- | --- | --- | --- | --- |
| 1 | 0 | 7.9 | 0 | 80 |
| 2 | 1 | 9.4 | 0 | 80 |
| 3 | 0.5 | 8.6 | 0.866 | 97 |
| 4 | -1 | 6.4 | 0 | 80 |
| 5 | -0.5 | 7.2 | -0.866 | 63 |
| 6 | 0.5 | 8.6 | -0.866 | 63 |
| 7 | -0.5 | 7.2 | 0.866 | 97 |

**
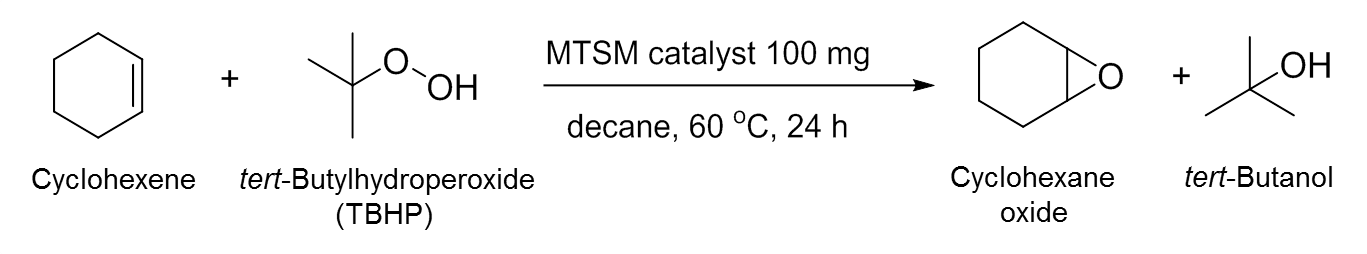
**

Scheme 1. Reaction of cyclohexene with TBHP under experimental conditions.

1. **Calibration curves, GC analysis and XPS data**

**Figure S4.** TBHP calibration curve.

**Figure S5.** Cyclohexene oxide calibration curve.

**Figure S6.** Cyclohexene calibration curve.


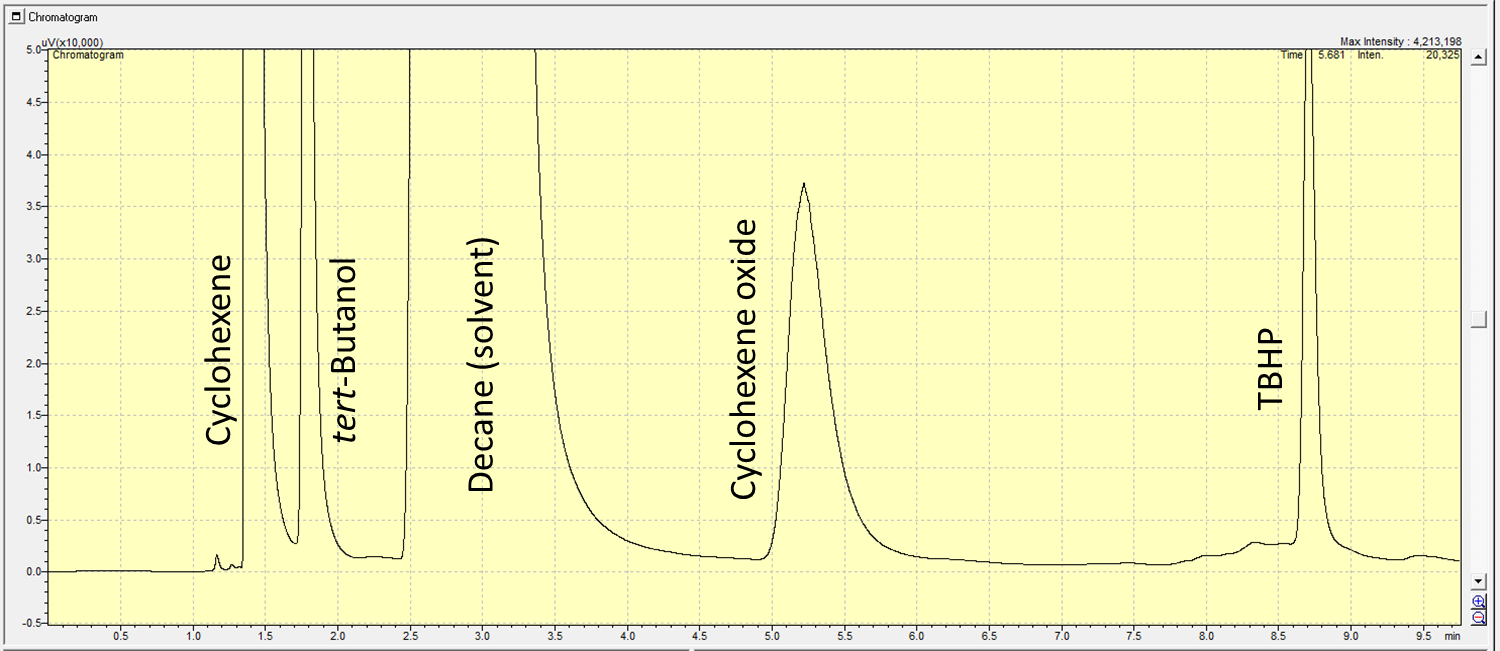


**Figure S7.** Gas chromatogram of reaction mixture, using DM1, sample 3 as catalyst, after 6 h of reaction.

1. **Details on calculations for catalytic experiments in Table 2**

Conversion of cyclohexene^a^ or TBHP^b^ (*x*):

$Conversion\left( x \right)=\left( 1- \left[ \frac{N_{x\left( t=t \right)}}{N_{x\left( t=0 \right)}} \right] \right) x 100\%$

*N_x_* = number of moles of *x*,

*t* = time

Yield of epoxide with respect to TBHP^c^:

$$Yield=\left[ \frac{N_{a\left( t=t \right)}}{N_{x\left( t=0 \right)}} \right] x 100\%$$

*N_a_* = number of moles of epoxide, *N_x_* = number of moles of TBHP

*t* = time

Epoxide selectivity (%)^d^:

Selectivity (%) = [moles of epoxide formed / total moles of TBHP reacted] x 100

1. **XPS and ICP data**

Table S2. XPS data for samples 1-13 and ICP data for samples 1-7.

| **Sample** | **Ti 2p _1/2_ / %** | **Ti 2p _3/2_ / %** | **Ti 2p total / %** | **Si 2p / %** | **O 1s / %** | **ICP Ti % in 1g of sample** |
| --- | --- | --- | --- | --- | --- | --- |
| 1 | 0.74 | 1.47 | 2.21 | 25.9 | 71.89 | 5.74 ± 0.29 |
| 2 | 1.15 | 2.3 | 3.45 | 29.35 | 67.19 | 5.04 ± 0.25 |
| 3 | 1.7 | 3.39 | 5.09 | 26.26 | 68.66 | 5.84 ± 0.29 |
| 4 | 0.83 | 1.65 | 2.48 | 29.37 | 68.15 | 4.86 ± 0.24 |
| 5 | 0.46 | 0.92 | 1.38 | 29.08 | 69.54 | 5.63 ± 0.56 |
| 6 | 1.43 | 2.85 | 4.28 | 26.81 | 68.9 | 6.55 ± 0.33 |
| 7 | 1.09 | 2.17 | 3.26 | 25.9 | 68.3 | 5.14 ± 0.26 |
| 8 | 1.26 | 2.51 | 3.77 | 28.45 | 67.99 | - |
| 9 | 0.75 | 1.49 | 2.24 | 28.24 | 68.01 | - |
| 10 | 0.97 | 1.94 | 2.91 | 29.75 | 67.86 | - |
| 11 | 1.06 | 2.11 | 3.17 | 29.23 | 68.33 | - |
| 12 | 1.72 | 3.42 | 5.14 | 26.07 | 68.8 | - |
| 13 | 1.27 | 2.54 | 3.81 | 27.68 | 68.52 | - |

1. TOF calculations for sample 7, using total Ti

Table S3. TOF values calculated using total Ti of sample 7, with respect to TBHP conversion, for different reaction times.

| **Reaction time / h** | **TOF for total Ti / h^-1^** |
| --- | --- |
| 2 | 52 |
| 4 | 42 |
| 6 | 33 |
| 8 | 26 |
| 24 | 4 |

1. **Doehlert matrix experiments for chemical structure modification: change of TEOS concentration and temperature**

**Table S4.** Doehlert matrix 3 (DM3): Change of TEOS concentration and temperature.

| **Sample** | **Factor 1** | **TEOS conc. / molL^-1^** | **Factor 2** | **Temperature / ⁰C** |
| --- | --- | --- | --- | --- |
| 1 | 0 | 2.35 | 0 | 80 |
| 2 | 1 | 2.6 | 0 | 80 |
| 3 | 0.5 | 2.48 | 0.866 | 97 |
| 4 | -1 | 2.1 | 0 | 80 |
| 5 | -0.5 | 2.2 | -0.866 | 63 |
| 6 | 0.5 | 2.48 | -0.866 | 63 |
| 7 | -0.5 | 2.2 | 0.866 | 97 |


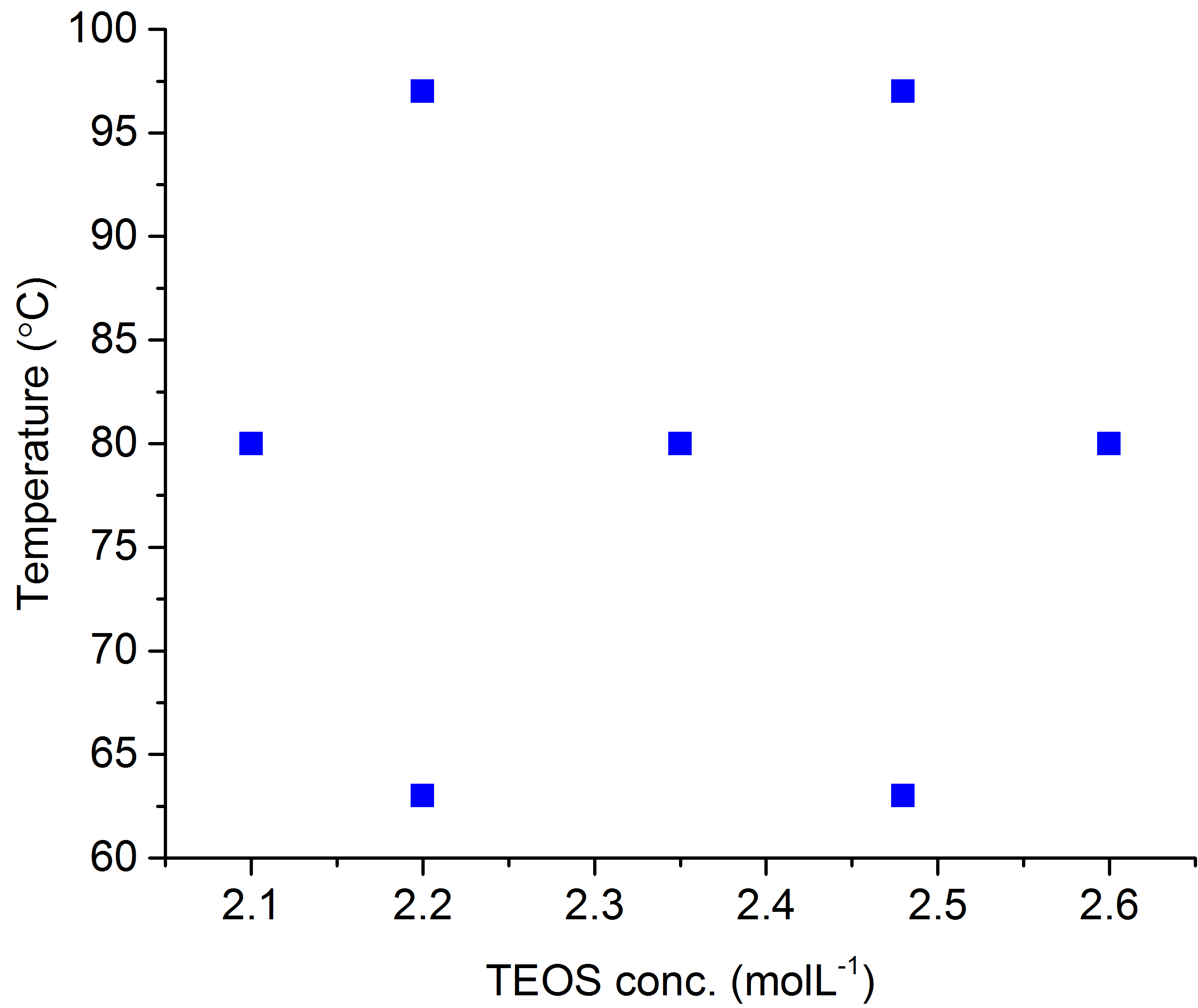


**Figure S8.** Samples 1-7, corresponding to Doehlert matrix 3 (DM3).

1. **EDX analysis**

Processing option: All elements analysed (normalized), number of iterations = 5

Standards: C: CaCO_3_ 1-Jun-1999 12:00 AM, O: Wollastonite (Si + Ca) 27-Sep-2010 01:34 PM, Si: Wollastonite (Si + Ca) 27-Sep-2010 01:33 PM, Ti: Rutile (Ti) 27-Sep-2010 01:16 PM

**Table S5.** EDX elemental analysis for DM1, sample 3.

| **Element** | **Weight %** | | **Atomic %** |
| --- | --- | --- | --- |
| C K | 20.86 | | 30.47 |
| O K | 45.57 | | 49.98 |
| Si K | 28.10 | | 17.55 |
| Ti K | 5.48 | | 2.01 |
| Totals | 100.00 | | 100.01 |
| 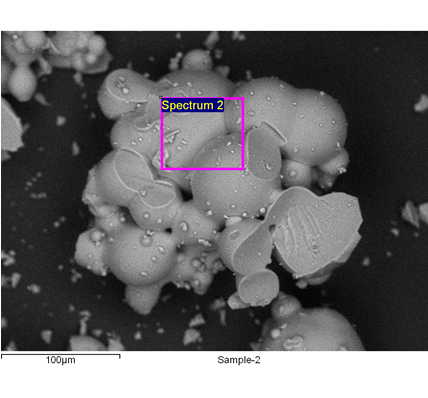 | | 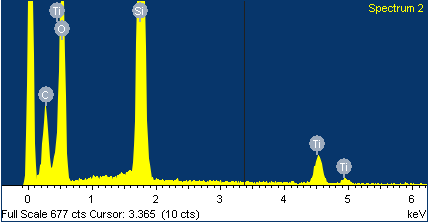 | |

**Figure S9.** Left - SEM image of sample 3 (DM 1), right - EDX spectrum.

1. **HRTEM imaging**


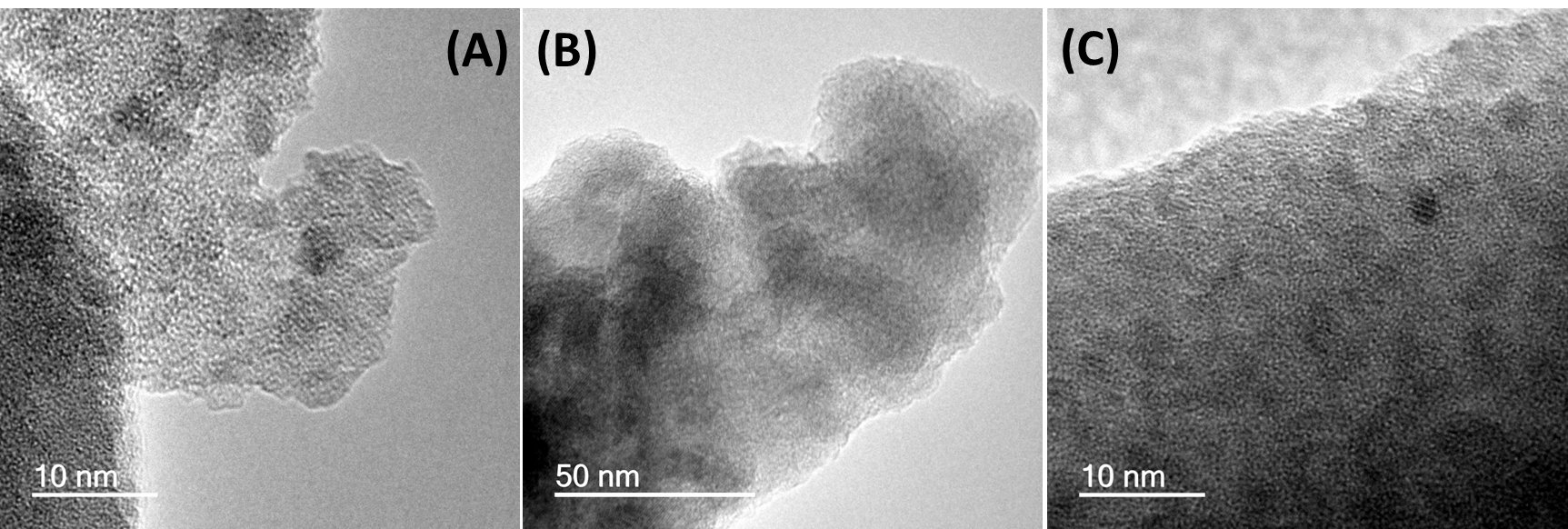


**Figure S10.** HRTEM images of MTSM: (A) sample 2, (B) sample 3 and (C) sample 7.

**Nitrogen physisorption data for samples 2, 3, 4 and 5**


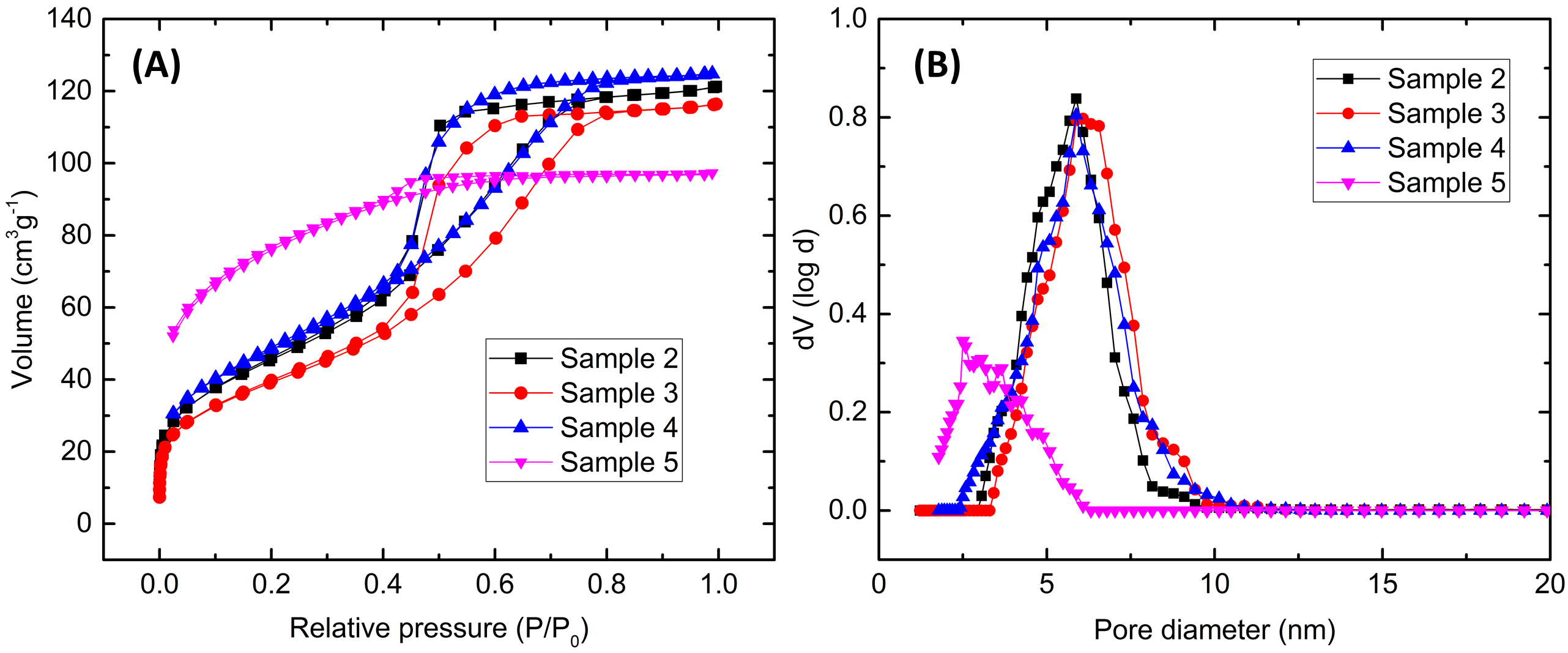


**Figure S11.** (A) Adsorption-desorption isotherms for samples 2, 3, 4 and 5, using N_2_ at 77K. (B) pore size distributions for samples 2, 3, 4 and 5, calculated using the NLDFT method.

**11. References**

1. S. L. C. Ferreira, W. N. L. dos Santos, M. A. Bezerra, V. A. Lemos and J. M. Bosque-Sendra, *Anal. Bioanal. Chem.*, 2003, **375**, 443–449.

2. D. H. Doehlert, *Applied Statistics*, 1970, **19**, 231–239.
